# Correction: Jonsdotter et al. MerTK and the Role of Phagoptosis in Neonatal Hypoxia-Ischemia. *Cells* 2025, *14*, 1862

**DOI:** 10.3390/cells15070621

**Published:** 2026-03-31

**Authors:** Andrea Jonsdotter, Henrik Hagberg, Anna-Lena Leverin, Joakim Ek, Kerstin Ebefors, Eridan Rocha-Ferreira, Ylva Carlsson

**Affiliations:** 1Department of Obstetrics and Gynecology, Sahlgrenska University Hospital, 416 85 Gothenburg, Västra Götaland, Sweden; henrik.hagberg@obgyn.gu.se (H.H.); ylva.carlsson.2@obgyn.gu.se (Y.C.); 2Centre of Perinatal Medicine & Health, Institute of Clinical Sciences, Sahlgrenska Academy, University of Gothenburg, 405 30 Gothenburg, Västra Götaland, Sweden; anna-lena.leverin@neuro.gu.se (A.-L.L.); joakim.ek@neuro.gu.se (J.E.); eridan.rocha.ferreira@gu.se (E.R.-F.); 3Centre of Perinatal Medicine & Health, Institute of Neuroscience and Physiology, Sahlgrenska Academy, University of Gothenburg, 405 30 Gothenburg, Västra Götaland, Sweden; 4Department of Physiology, Institute of Neuroscience and Physiology, Sahlgrenska Academy, University of Gothenburg, 405 30 Gothenburg, Västra Götaland, Sweden

## Missing Citation

In the original publication [1], ref. [17] were not cited. The citation has now been inserted in Caspase-3 Activity Measurement, and should read as follows:

Caspase-3 activity was measured using a fluorometric DEVD-AMC assay (Substrate #3171v, Peptides International, Louisville, KY, USA) according to a protocol previously established and validated in our laboratory (Wang et al., 2001) [17].

## Text Correction

There was an error in the original publication. The correction concerns the methodological description of the caspase-3 activity assay. In the original manuscript, the wording unintentionally implied that a manufacturer-provided standard protocol had been used (“Caspase-3 activity was measured using a standard fluorometric DEVD-AMC assay according to the manufacturer’s instructions”). This was inaccurate, as all experiments were in fact performed according to a protocol previously established and validated in our own laboratory.

A correction has been made to Caspase-3 Activity Measurement, as follows:

Caspase-3 activity was measured using a fluorometric DEVD-AMC assay (Substrate #3171v, Peptides International, Louisville, KY, USA) according to a protocol previously established and validated in our laboratory (Wang et al., 2001) [17].

## References

Wang, X.; Karlsson, J.O.; Zhu, C.; Bahr, B.A.; Hagberg, H.; Blomgren, K. Caspase-3 activation after neonatal rat cerebral hypoxia-ischemia. *Biol. Neonate* **2001**, *79*, 172–179. http://doi.org/10.1159/000047087.

With this correction, the order of some references has been adjusted accordingly. The authors state that the scientific conclusions are unaffected.

This correction was approved by the Academic Editor. The original publication has also been updated.

## References

[1] Jonsdotter A., Hagberg H., Leverin A.-L., Ek J., Ebefors K., Rocha-Ferreira E., Carlsson Y. (2025). MerTK and the Role of Phagoptosis in Neonatal Hypoxia-Ischemia. Cells.

